# Missed vaccine opportunities for *Streptococcus pneumoniae* and influenza in patients admitted during the coronavirus disease 2019 (COVID-19) pandemic

**DOI:** 10.1017/ice.2020.1294

**Published:** 2020-10-26

**Authors:** Anita Shallal, Rachel Kenney, Allison Weinmann

**Affiliations:** 1 Division of Infectious Diseases, Department of Internal Medicine, Henry Ford Hospital, Detroit Michigan; 2 Department of Pharmacy Services, Henry Ford Hospital, Detroit, Michigan; 3 Antimicrobial Stewardship, Division of Infectious Diseases, Henry Ford Health System, Detroit, Michigan; 4 Office of Clinical Quality and Safety, Henry Ford Health System, Detroit, Michigan; 5 Department of Internal Medicine, Wayne State University, Detroit, Michigan

*To the Editor*—Detroit, Michigan, and its surrounding counties emerged as a hot spot for the coronavirus disease-2019 (COVID-19) pandemic in March and April of 2020. A number of hospitalized patients had numerous comorbidities, which increased the risk of acquiring the infection: age >65 years, heart or lung disease, lung disease, and diabetes. These comorbidities are also associated with other infections, including invasive *Streptococcus pneumoniae* infection. Our institution follows a standing order for nurses to vaccinate adults aged >65 years who meet the Advisory Committee on Immunization Practices (ACIP) criteria for the 23-valent pneumococcal polysaccharide vaccine (PPSV23) to reduce *S. pneumoniae* infections^1,2^ and against influenza during flu season. During the COVID-19 pandemic surge, the pneumococcal vaccine and influenza vaccine nurse-driven protocols were determined to be nonessential on March 23, 2020, and April 2, 2020, respectively. In this study, we aimed to characterized missed vaccine opportunities among patients hospitalized with COVID-19 during this surge.

We performed a cross-sectional study of PCR-positive COVID-19 patients admitted to an inner-city tertiary-care health system and discharged alive between the dates of March 23 and April 21, 2020. Patients under the age of 65 were excluded. Data were collected retrospectively and included patient age, gender, race, length of stay, comorbidities that would indicate a vaccine opportunity, prior vaccinations, and whether there was a vaccine opportunity for PPSV23 and influenza defined by ACIP indications.^2^ The indications included age >65 years, chronic lung disease, chronic kidney disease, cardiomyopathy or heart failure, human immunodeficiency virus (HIV), solid-organ malignancy or multiple myeloma, immunosuppressed (ie, on immunosuppressing drugs, long-term steroids, or solid-organ recipient), and other (eg, cochlear implant, cerebrospinal fluid leak, post-splenectomy, sickle-cell disease, or alcohol-use disorder). Vaccine history was evaluated using the electronic medical record (EMR) and Michigan Care Improvement Registry (MCIR). If there was a vaccine opportunity, we documented whether or not a vaccine was given prior to hospital discharge. In addition, the total numbers of vaccines given for the same periods in 2019 and 2020 were collected from the EMR for comparison. Descriptive analysis was utilized.

Overall, 100 patients over the age of 65 were included. The median age was 71 years; most patients (66%) were of African American race; and 87% of patients received antibiotics during hospitalization. We identifed 52 patients as having an opportunity to receive PPSV23, and none patients received the vaccine. Furthermore, 37 patients were eligible to receive influenza vaccine, and none received the vaccine. The median length of stay was 4 days, and 18 patients were readmitted within 30 days.^3,6^ Additional results are summarized in Table 1. According to the EMR, the total numbers of pneumococcal vaccinations given per nurse-driven protocol at our institution were 238 for March, 216 for April, and 218 for May. However, these numbers dropped to 123 for March, 11 for April, and 29 for May, corresponding to a percentage decrease in the number of vaccinations given to between 48% and 94%. Similarly, the total numbers of influenza vaccinations given per nurse-driven protocol at our institution were 399 for March, 228 for April, and 38 for May. For the same period in 2020, these numbers dropped to 316 for March, 14 for April, and 0 for May, corresponding to a percentage decrease again as high as 94%.

**Table 1.** Summary of Results

Category	No. (%)
Age, median y (IQR)	71 (69–76)
Sex, male	46 (46)
**Race**	
African American	66 (66)
Caucasian	6 (6)
Hispanic or Latino	1 (1)
Unknown	27 (27)
Prior vaccination with PPSV23 or PCV13	57 (57)
**Indications for vaccine apart from age >65 y**	
Heart failure or cardiomyopathy	44 (44)
Chronic kidney disease	31 (31)
Chronic lung disease	15 (15)
Solid organ malignancy or multiple myeloma	4 (4)
Immunosuppressed^a^	4 (4)
Other^b^	3 (3)
Liver disease	2 (2)
HIV positive	1 (1)
**Vaccine opportunity**	
*Streptococcus pneumoniae* (PPSV23)	52 (52)
Influenza vaccine	37 (37)
**Vaccine given** *S. pneumoniae* (PPSV23) Influenza vaccine	0 (0) 0 (0)
**No. of indications per ACIP guidelines for** ***S. pneumoniae*** **vaccine among those with missed vaccine opportunity**	
1 indication (age >65 y only)	17/52 (32)
2 indications	23/52 (44.2)
3 indications	8/52 (15.4)
4 or more indications	4/52 (7.7)

Note. IQR, interquartile range; HIV, human immunodeficiency virus; ACIP, Advisory Committee on Immunization Practices.

a Immunosuppressed: on immunosuppressing drugs, long term steroids, or solid-organ transplant recipient

b Other: cochlear implant, CSF leak, post-splenectomy, sickle cell disease, or alcohol use disorder.

Due to prioritization of potential staffing shortages and clustering nursing care, a nursing-by-designated-authority protocol for immunization was deemed nonessential during the COVID-19 pandemic surge. As a result, opportunities to vaccinate patients with pneumococcal and influenza vaccines were missed despite an average hospitalization of 4 days to provide the vaccine(s). Of the 52 patients who had an opportunity for pneumococcal vaccination, most (67.3%) had >1 indication for PPSV23, with 7.7% having 4 or more indications. These patients, due to numerous comorbidities, are at high risk for severe pneumococcal disease. The Centers for Disease Control and Prevention (CDC) has offered guidance that during the pandemic, confirmed positive COVID-19 patients should defer routine immunization.^3^ Although a febrile illness is not a direct contraindication to receiving an inactivated vaccine, the CDC advises that for hospitalized patients with moderate-to-severe illness, routine immunization can be delayed until time of hospital discharge or until isolation precautions are discontinued.^2^ The resasoning is not due to concern for reduced vaccine efficacy nor vaccine adverse effects, but rather the potential adverse events that may complicate treatment of the moderate-to-severe illness.^2^ We argue that, provided appropriate personal protective equipment in use, the benefits of preventing invasive pneumococcal disease and severe influenza potentially outweigh the risks associated with vaccination, and it is appropriate to cluster such a measure with other nursing-care activities at the time of hospital discharge.

Routine immunization programs across the world are now at risk due to health system constraints, shelter-in-place orders, and physical-distancing measures.^4^ In addition, many patients avoid seeking health care due to fear of contracting COVID-19. It is important for healthcare providers to be aware of all missed vaccine opportunities, especially in high-risk patients with moderate-to-severe COVID-19, to facilitate disease prevention and improve vaccine coverage. Preventing other infections, such as influenza or *S. pneumoniae*, is a form of primary prevention that can also help reduce the burden on the healthcare system during pandemic surges. On a larger scale, deeming effective immunization programs as nonessential may simply delay disease, hospitalizations, and death in at-risk patients.
